# Wrist accelerometry for monitoring dementia agitation behaviour in clinical settings: A scoping review

**DOI:** 10.3389/fpsyt.2022.913213

**Published:** 2022-09-16

**Authors:** James Chung-Wai Cheung, Bryan Pak-Hei So, Ken Hok Man Ho, Duo Wai-Chi Wong, Alan Hiu-Fung Lam, Daphne Sze Ki Cheung

**Affiliations:** ^1^Department of Biomedical Engineering, Faculty of Engineering, The Hong Kong Polytechnic University, Kowloon, Hong Kong SAR, China; ^2^Research Institute for Smart Ageing, The Hong Kong Polytechnic University, Kowloon, Hong Kong SAR, China; ^3^The Nethersole School of Nursing, The Chinese University of Hong Kong, Shatin, Hong Kong SAR, China; ^4^Department of Electrical Engineering, City University of Hong Kong, Kowloon, Hong Kong SAR, China; ^5^School of Nursing, The Hong Kong Polytechnic University, Kowloon, Hong Kong SAR, China

**Keywords:** Alzheimer's disease, agitation, wearable device, dementia, wandering, wristband, mild cognitive impairment, aggression

## Abstract

Agitated behaviour among elderly people with dementia is a challenge in clinical management. Wrist accelerometry could be a versatile tool for making objective, quantitative, and long-term assessments. The objective of this review was to summarise the clinical application of wrist accelerometry to agitation assessments and ways of analysing the data. Two authors independently searched the electronic databases CINAHL, PubMed, PsycInfo, EMBASE, and Web of Science. Nine (*n* = 9) articles were eligible for a review. Our review found a significant association between the activity levels (frequency and entropy) measured by accelerometers and the benchmark instrument of agitated behaviour. However, the performance of wrist accelerometry in identifying the occurrence of agitation episodes was unsatisfactory. Elderly people with dementia have also been monitored in existing studies by investigating the at-risk time for their agitation episodes (daytime and evening). Consideration may be given in future studies on wrist accelerometry to unifying the parameters of interest and the cut-off and measurement periods, and to using a sampling window to standardise the protocol for assessing agitated behaviour through wrist accelerometry.

## Introduction

Agitation is highly prevalent among older adults with dementia, ranging from 24.8 to 71% depending on the severity of the dementia (1–4). Individuals have exhibited subsyndromes of dementia, including agitation, psychosis, affective symptoms, and apathy, with the level of agitation increasing with the severity of the dementia (5). Healthcare providers may find it challenging in caring agitated people with dementia in places such as homes, hospitals, residential care homes, and elderly day centres (6). Safety issues are significant considerations in elderly care. Agitation-induced wandering behaviour or physical aggression increases the fall and injury risks for the agitated individuals, the people around them, and caregivers, and are associated with an increased use of restraints, both physical (e.g., bedside rails, alarm pads) and chemical (e.g., antipsychotic and hypnotic drugs). These restraints might in turn aggravate psychosocial and agitation problems among older adults (7), precipitating stress, embarrassment, and safety risks among informal caregivers and reducing their quality of life (8, 9). Family caregivers might then send their relatives to residential care homes, increasing the healthcare burden.

Agitated behaviour is broadly classified as excessive motor activity, verbal aggression, or physical aggression by patients with cognitive impairment and emotional distress, which may cause excess disability (10). Agitated behaviour is not solely attributable to the care environment, other disorders, or to the effects of taking a substance (10). It could also be found in individuals without cognitive impairment (11). Non-aggressive physical behaviours include wandering, hiding things, and repetitive mannerisms. Individuals may present verbally, through such behaviours as complaining, asking repetitive questions, and making unwarranted requests. Aggressive behaviours include screaming noises, which could advance to aggressive physical actions such as throwing things, hitting, and hurting oneself or others.

Traditionally, agitation is identified *via* informant rating and observation. Informant ratings are based on the frequency and severity of the agitated behaviours reported by caregivers, although these are subjective, prone to bias, and blended with the ratings for other dementia symptoms (12, 13). An observational method has been proposed, involving videotaping an individual's behaviour at a given time and having trained personnel watch the videotape (12). Apart from observer bias, a long duration of observation is required to detect low-frequency agitated behaviours, making continuous monitoring infeasible (6, 12).

Technological assessments enable objective, manpower-saving, and continuous monitoring of agitated behaviours, while traditional observational approaches or questionnaires are generally subjective. Actigraphy or accelerometry is increasingly being applied to monitor agitation through the measurement of physical activity among older adults (14). Actigraphy and accelerometry are often used interchangeably (15). While accelerometers refer to the sensors that measure acceleration of body segments (16), actigraphy is a process that involves applying accelerometer data to quantify and infer motor or behavioural activities, such as sleeping and waking, often through a wrist-worn accelerometric measurement device (17). Accelerometers detect movement by the acceleration and deceleration signals induced by the initialisation and termination of movements. There is no acceleration when the movement of the body is steady or stationary. The principle of assessment lies in the characteristics of repetitive movements when agitated, such as restlessness and pacing (18). Accelerometers, some integrated with gyroscopes, can detect movements and changes of velocity at a wide range of frequencies. The collected data can be used to classify different postures, activities, postural transitions, and walking patterns (19, 20), and has been used to evaluate wandering behaviour (21), restlessness during sleep (22), physical exercise levels (23), walking instability (24), and falling events (25). Moreover, the validity of accelerometry in agitation assessments has been demonstrated through correlation with other instruments, including the Cohen-Mansfield Agitation Inventory (CMAI) and the Neuropsychiatric Inventory (NPI) (26). However, its application and analytical methods have varied across studies, which may have hindered its usage and the development of related research.

Healthcare assessments and management for older adults in clinical settings remain one of the greatest challenges in public health, especially for those with dementia or other chronic diseases. Common behavioural symptoms associated with dementia, such as agitation and wandering, impose significant psychological distress on caregivers and fall or injury risks to older people. The coronavirus disease 2019 (COVID-19) pandemic has led to pressure on healthcare workers and staff shortages. In addition, social distancing and mask-wearing measures have negatively impacted older people and potentially exaggerated the behavioural problems of those with dementia (27–29). Healthcare technology, such as wrist accelerometry for agitation monitoring, provides a versatile platform for real-time and prolonged assessments to facilitate better clinical management and relieve the staffing and hospitalisation burden (30). Moreover, traditional screening or assessment methods rely on subjective observations from professionals or data from questionnaires. Wrist accelerometry may provide an objective way to evaluate and quantify levels of agitated behaviour.

There have been some similar reviews of this topic. Camargos et al. (31) focused on the application of wrist accelerometry in night-time agitation and sleep disturbances in comparison to polysomnography, while Cheung et al. (19) reported on the application of accelerometry in determining daily activities. Khan et al. (6) endeavoured to summarise platforms for automatic detection and generalised predictive models for sensors in detecting agitation and aggression. The objectives of this review are to supplement previous reviews by: (1) reviewing the application of wrist accelerometry for monitoring agitated behaviours among people with dementia in clinical settings; (2) understanding the analytical approach of wrist accelerometry in assessing agitated behaviours; and (3) commenting on the potential weaknesses of current applications.

## Methods

### Design

This scoping review was based on the approach of Arksey and O'Malley (32), which includes five steps: (i) identifying the research question; (ii) searching for relevant studies; (iii) selecting the studies; (iv) charting the data; and (v) collating, summarising, and reporting the results. The Preferred Reporting Items for Systematic Reviews and Meta-Analyses Extension for Scoping Reviews (PRISMA-ScR) guidelines were followed in reporting the study (33).

### Step 1: Identify research questions

1. How was the wrist accelerometry/actigraphy applied in the clinical settings to monitor agitation for individuals with dementia?
2. How was wrist accelerometry/actigraphy evaluated by comparing the results to those obtained with a benchmarked instrument or by comparing the results of individuals with and without agitation?
3. How were the collected data analysed?

### Step 2: Searching for relevant studies

#### Eligibility

Eligibility was limited to original research articles published in peer-reviewed academic journals in English from the year 2000 onwards, a time during which the application of accelerometry for agitation monitoring had gained acceptance (14). The inclusion criteria included: (1) studies in which the participants had been diagnosed with any type of dementia (such as Alzheimer's disease or vascular dementia); (2) a study design that included the application of a wrist accelerometer/actigraph for monitoring agitation, an investigation of those with agitated and non-agitated dementia, and validation against/association with benchmarked instruments for measuring agitation; (3) studies conducted in non-laboratory clinical field settings, such as communities, hospitals, or residential care homes.

Exclusion criteria included: (1) non-original research (e.g., reviews or perspective articles); (2) articles not from academic journals (e.g., conference papers, patents); (3) articles with insufficient content on the setting and results of the accelerometry on dementia agitation: (4) studies where the accelerometry/actigraph was not on a wrist configuration; (5) studies not primarily dedicated to or measuring agitation (e.g., measuring rest and activity levels without addressing agitation); (6) intervention studies (e.g., those evaluating the influence of a medication or therapy).

#### Information sources and search strategy

A systematic literature search was performed to identify studies reporting on the application of wrist accelerometry in monitoring the agitated behaviour of people with dementia. The literature search was performed on databases including Web of Science, PubMed, PsycInfo, EMBASE, and CINAHL. The first and second author ran the independent searches on 20 March 2022. The search was conducted using MeSH or natural language keywords in the title and abstract of articles related to dementia, accelerometry/actigraphy, and agitation. The full search strategy is set out in Supplementary Table 1.

### Step 3: Selecting the study

Titles and abstracts were screened first, followed by a full-text screening according to the eligibility criteria. The two-step screening process was conducted by the first and second authors working independently. Any disagreements were resolved by seeking a consensus with the corresponding author. To ensure the consistency between two reviewers, they had pilot reviewed 10% of the retrieved articles at both stages according to the selection criteria and got a consensus before reviewing separately.

### Step 4: Data charting

A data charting form was designed based on the research questions. Information on the studies was extracted, including a summary of background information on the study, accelerometer/actigraph measurements, data analyses, and findings. The data charting results were cross-checked to ensure accuracy.

### Step 5: Collating, summarising, and reporting the results

A descriptive summary was developed to collate and summarise the results.

## Results

### Search results

**Figure 1:**
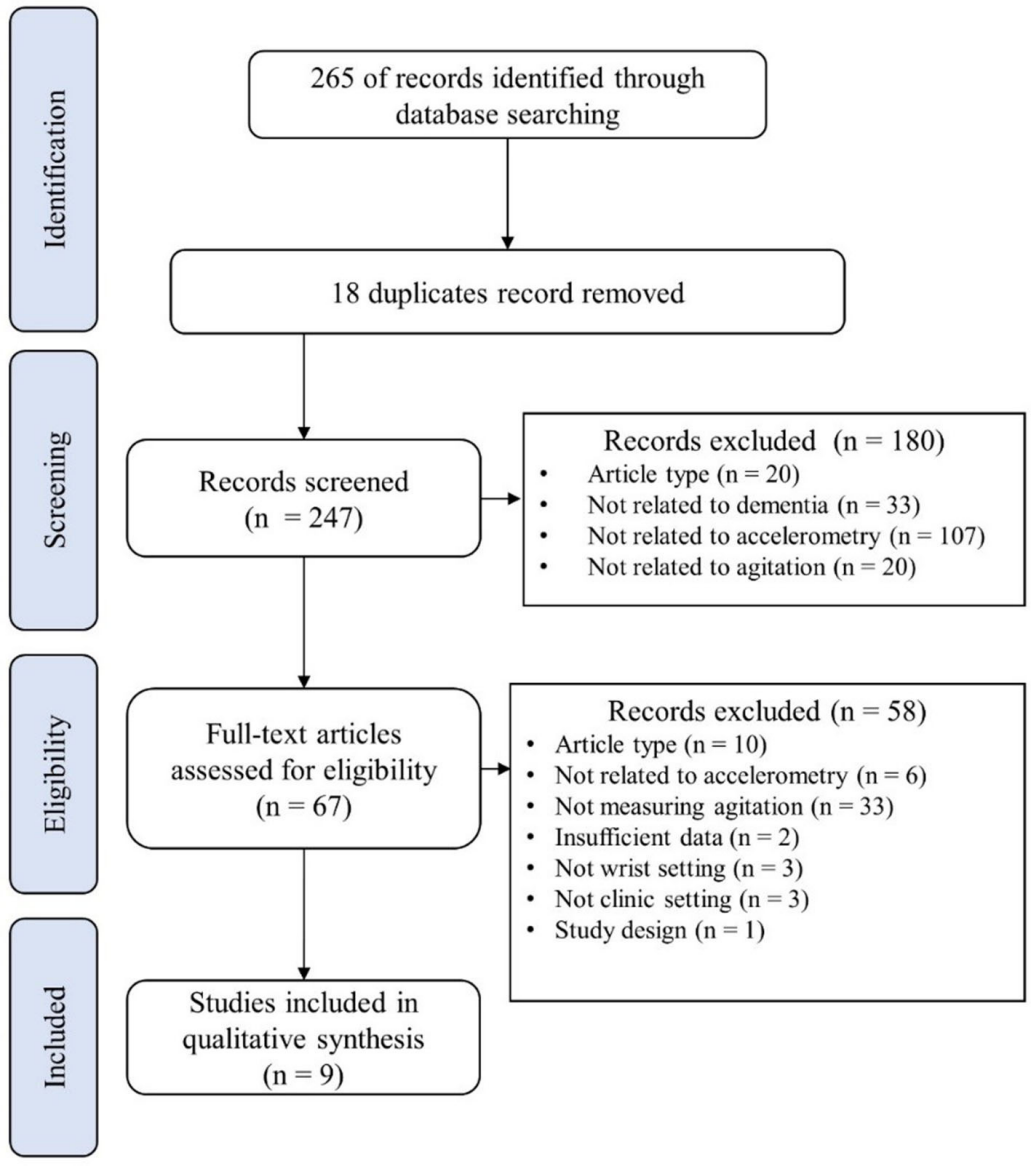
Flowchart of the systematic search and screening.

An initial search yielded 333 articles, from which 49 duplicates were removed. A preliminary screening of titles and abstracts was conducted on 284 records, and 186 of them were excluded because the articles were irrelevant (*n* = 2), of the wrong type (e.g., review articles, conference papers) (*n* = 20), not in English (*n* = 1), unrelated to accelerometer/actigraph (*n* = 107), agitation (*n* = 20), or dementia (*n* = 35), or not a human study (*n* = 1). Ninety-eight articles remaining after the preliminary screening, the full texts of which were then screened. Eighty-nine of them were excluded, the reasons being that the article did not appear in a journal (*n* = 1), was of the wrong type (*n* = 8), contained insufficient data (*n* = 2), was an intervention study (*n* = 17), not conducted in a clinical settings (*n* = 5), the study design was ineligible (*n* = 7), the study was not related to or did not measure agitation (*n* = 29), was not related to or did not measure agitation by accelerometer/actigraph (*n* = 16), and the accelerometer/actigraph was not at a wrist setting (*n* = 4). In the end, nine articles were eligible for the review. Figure 1 shows the flowchart of this selection process.

### Study characteristics

Although the parameters of the literature search included articles published as early as 2000, the earliest article found was published in 2006. Among the nine articles, five were on studies conducted in the Americas, three in Europe, and one in Asia. As shown in Table 1, the articles involved 373 individuals (134 males and 239 females), with sample sizes ranging from 1 to 183. Six out of nine articles had a sample size of <20. Nearly all of the participants were older, with a pooled weighted average age of 82. Most of the studies involved participants with Alzheimer's disease or other types of dementia. They were diagnosed using the National Institute of Neurological Disorders and Stroke-Alzheimer Disease and Related Disorders (NINCDS-ADRDA) criteria (42), measurements drawn from the *Diagnostic and Statistical Manual of Mental Disorders* (43) or by the Clinical Dementia Rating Scale (44).

**Table 1:** Basic information on the eligible articles.

**Article**	**Sample size**	**Sample**	**Age (range)**	**Country**	**Field setting**	**Placement**	**Duration**
Au-Yeung et al. (34)	1 M	AD	64	United States	Memory care facilities	N-Dm wrist	138 days
Bankole et al. (35)	6 F	DE	81.8 (67 to 93)	United States	Long-term dementia care unit	Dm wrist, waist, opposite leg	28 days
Goerss et al. (36)	11 F/6 M	DE	^*^83.5 (73 to 94)	Germany	Nursing homes	Dm wrist, right ankle	28 days
Ishimaru et al. (37)	8 M 6 M	Sev. DE Mod. DE	90.2 (SD: 6.4) 87.5 (SD: 6.1)	Japan	Hospital	N-Dm wrist and Dm-hand (if paresis)	3 to 6 days
Knuff et al. (26)	4 F/16 M	AD/related dementias	74.3 (8.69)	Canada	Inpatient unit and long-term care facility	N-Dm wrist	1 to 7 days
Nagels et al. (38)	65 F/45 M	Vascular DE	78 (SD: 8)	Belgium	Memory clinic	N-Dm wrist	2 days
Spasojevic et al. (39)	10 F/7 M	Agitated behaviour	78.88 (SD: 8.86, 65 to 93)	Canada	DE unit	-	Max 60 days
Valembois et al. (40)	140 F/43 M	-	84.9 (SD: 6.8)	France	Intermediate care unit	N-Dm wrist	10 days
Wijbenga et al. (41)	3 F/2 M	DE	^*^83.5 (78 to 89)	United States	Nursing homes	-	63 days

* Information on average age was not included and was estimated by the average of the range.

AD, Alzheimer's disease; Ctrl, Control; DE, Dementia; Dm, Dominant; F, Females; M, Males; N-Dm, Non-dominant; SD, Standard deviation.

Most of the work took place in specialised care units, in addition to nursing homes and inpatient wards. The measurement periods ranged from 2 days to 138 days and five out of nine studies had a measurement period of more than 28 days (i.e., 4 weeks). Some studies did not employ a unified measurement time for their participants (26, 37, 39). Six included studies placed the accelerometer device on the non-dominant wrist, while two studies placed it on the dominant wrist. One study emphasised that the accelerometer device was switched to the dominant wrist if a participant had paresis on the non-dominant wrist (37). Another study mentioned that an ankle accelerometer was used during the night so that the battery could be recharged on the wrist accelerometer during the daytime (36). Two articles also included accelerometer measurements of the ankle or waist (35, 36).

Regarding the objectives or design of the studies, two articles implemented a descriptive approach to dementia with agitation samples, and then investigated the relationship between agitated episodes and some risk factors (34, 41). Six studies correlated the accelerometric data to a benchmarked instrument and one of them further evaluated the differences between individuals with different levels of agitation (26). Valembois et al. (40) compared the accelerometry between individuals with and without agitation, whilst Spasojevic et al. (39) evaluated the internal validity of different classifiers on identifying agitation episodes.

### Accelerometric measurement

The accelerometric devices that were used in the studies covered in the review articles differed (see Table 2). All but one study applied commercially available accelerometric or actigraphic devices. These included Actiwatch Spectrums (Philips Respironics, Murrysville, United States), Grey Innovation Sensors (Grey Innovation, Melbourne, Australia), Micro Motionlogger WatchWare (Ambulatory Monitoring Inc., New York, United States), wGT3x+ (ActiGraph LLC, Pensacola, United States), Actiwatch (Neurotechnology Co., Cambridge, United Kingdom), Empatica E4 wristband (Empatica Inc., Boston, United States), Vivago (Vivago Oy, Espoo, Finland), and MotionWatch 8 (CamNtech, Cambridgeshire, UK). It should be noted that some of these devices incorporated gyroscopes and could be called inertial measurement units (IMUs). On the other hand, Bankole et al. (35) utilised the concept of the body sensor network using a collection of wearable sensor nodes (45), processing and integrating the data through technology-enabled medical precision observation (TEMPO) technology.

**Table 2:** Accelerometer specification, data conditioning, and endpoints.

**Article**	**Device (Type)**	**Software**	**Conditioning**	**Sampling approach**	**Measurement window**	**Endpoint/ parameter**
Au-Yeung et al. (34)	Actiwatch Spectrums (Piezoelectric)	Developed by ORCAT-ECH	-	Per 15 s epoch	Day: 6 am to 2 pm Evening: 2 pm to 10 pm Night: 10 pm to 6 am	Total activity counts
Bankole et al. (35)	Body Sensor Network (triaxial IMU)	TEMPO	-	2 min for event	Morning: 3 h Afternoon: 3 h Evening: 3 h	Teager energy
Goerss et al. (36)	Sensors from Grey Innovation (triaxial IMU)	-	Resampling Sinc bandpass philtre, signal rectification, Sinc low-pass philtre	100 Hz	Day: 8 am to 6 pm Night: 6 pm to 8 am	AMS per 10 s
Ishimaru et al. (37)	Micro Motionlogger Watchware	-	-	-	Day: 9 am to 5 pm Full-day	Amount of activity (daytime, total, hourly mean each day)
Knuff et al. (26)	wGT3x+ (triaxial MEMS)	-	-	-	Daytime: 6 am to 2 pm Evening: 2 pm to 10 pm Night-time: 10 pm to 6 am	Mean motor activity
Nagels et al. (38)	Octagonal Basic Motionlogger (Piezoelectric)	Java	Low-pass philtre	30 min epoch at epoch length of 1 s	Daytime: 9 am to 9 pm	ZCM, TAT, PIM
Spasojevic et al. (39)	Empatica E4 (triaxial MEMS)	-	Resampling, low-pass philtre	1, 3, and 5 min and aggregated the features in analysis	Full-day	Teager energy, Signal value, spectral entropy DC power
Valembois et al. (40)	Vivago	Bundled software	-	Per second	Full-day (every 3 h)	Mean motor activity every 3 h
Wijbenga et al. (41)	MotionWatch 8 (Triaxial)	MotionWare	-	Per 30 s epoch	Full-day	Activity counts

AMS, Accelerometric motion score; MEMS, Microelectromechanical systems; ORCAT-ECH, Oregon Centre for Ageing and Technology; PIM, Proportional Integrating Measures; TEMPO, Technology-enabled medical precision observation; TAT, Time-Above-Threshold; ZCM, Zero Crossing Mode.

Some studies employed an accelerometer with fewer than three axes (34, 38). Cheung et al. (19) recommended against this practise because the movements associated with agitation are not always in two dimensions. In addition, some of the devices employed in the included studies incorporated IMU, which is more desirable. This is because static acceleration, such as gravity, makes it impossible for an accelerometer to differentiate between dynamic translation and rotational motion (46). An accelerometer with a gyroscope and a magnetometer as an IMU can fully interpret motion signals. The integrated sensors demonstrate better reliability and accuracy and also enable the intensity of movements to be determined and thus allow activities to be classified (19). Two studies (34, 38) employed piezoelectric accelerometers in contrast to the ordinary accelerometers based on Microelectromechanical systems (MEMS) used in other studies. A piezoelectric accelerometer consists of a seismic mass hanged by a piezoelectric beam, which has undergone bending and has displaced charges upon acceleration (47).

Two-thirds of the studies did not provide adequate information on the data processing or filtering methods that were used, but some studies reported that they had utilised the stipulated bundled software for processing the data. While Goerss et al. (36) and Spasojevic et al. (39) resampled the data before processing it, three studies utilised different signal filtering strategies, including a low-pass filter at 2 to 3 Hz (38), a Butterworth low-pass filter at the 6 Hz cut-off point (39), and a Sinc bandpass and low pass philtre with signal rectification (36). Goerss et al. (36) defined the Accelerometric Motion Score (AMS) as the average activity count after data conditioning.

Nevertheless, the endpoint parameters for all studies were different. Therefore, comparing outcomes was difficult, especially when the sampling, measurement, and selection of averaging sums differed. The total amount and average amount of activity over a period were intuitive estimation and commonly used for evaluation (26, 34, 36, 37, 39–41). With the Zero Crossing Mode (ZCM) and the Time-Above-Threshold (TAT), the occurrence and time of the activity are taken into consideration only when the accelerometer signal is above the threshold value (38). Some other analyses recommended that at least 3 days of measurements are needed to accurately estimate physical activity levels (48). Relevant information is needed for assessing dementia agitation. In addition, two studies endeavoured to identify the occurrence of agitation episodes from labelled data, instead of recognising individuals with agitated behaviour in general (35, 39).

Apart from the time domain signal analytics (i.e., the number of counts), the frequency response of the triaxial accelerometer signals can also be analysed using the Fourier transform, Laplace transform, or other similar ways of identifying the peak of the amplitude and its frequency with regard to the 3-axis acceleration signal (49). On the other hand, by using power spectrum density (PSD), the power level of 3-axis acceleration signals and the resultant acceleration can be shown (50). The change in power level from a calm state to an agitated state may provide further data to shed light on the nature of the agitated behaviours.

Entropy-related parameters were also applied in some analyses. For example, the Proportional Integrating Measure (PIM) was calculated based on the area under the rectified accelerometer signal (38). The Teager energy score, which appeared in two articles, is a measure of aggregated movement energy and was calculated by the sum of the amplitude squared and the frequency squared of the signal (35, 39). Spasojevic et al. (39) integrated many parameters for detecting agitation, including the maximum, minimum, mean, and simple square integral of the Teager energy; and the mean, minimum, standard deviation, interquartile range, spectral entropy, and DC power of the accelerometer signal. A Pearson correlation analysis was conducted, with a cut-off point of 0.9 to avoid multicollinearity.

### Data analysis and key findings

The protocol for measuring activity differed among studies according to their research objectives. Three articles considered a global association of activity counts with agitation (e.g., summing the activity count over 24 h) (26, 37, 41), while Valembois et al. (40) also took measurements over 24 h but analysed the data every 3 h. Au-Yeung et al. (34) stratified the full-day measurement into days (6 am to 2 pm), evenings (2 pm to 10 pm), and nights (10 pm to 6 am). Two articles defined the daytime activities differently, from 9 am to 5 pm (37), and from 9 am to 9 pm (38), respectively. Goerss et al. (36) monitored a morning and an afternoon section, while Bankole et al. (35) picked three sets of 3-h sections at different times of the day.

Nearly all studies investigated the association between accelerometric-measured activity and measures of agitated behaviour, particularly using parametric tests (e.g., *t*-test, ANOVA) or non-parametric tests (e.g., Wilcoxon's signed-rank test, the Mann-Whitney U test) to compare groups with or without agitation; and correlation tests (e.g., Spearman or Pearson tests) between the level of agitation and accelerometry readings (see Table 3). Spasojevic et al. (39) constructed several classifiers to segregate agitation episodes with accelerometer signals, but all produced poor performance, with the Area under the Receiver Operating Characteristics (AUC) being <0.65.

**Table 3:** Data analysis and key findings.

**Article**	**Benchmarking**	**Classifier/Statistics**	**Findings**
Au-Yeung et al. (34)	Nursing note	*T*-test	Total activity counts: ↑ in daytime and night time in agitated shifts ⊗ evening time between agitated and non-agitated shifts
Bankole et al. (35)	CMAI, ABS, MMSE	Spearman correlation	In morning, Teager score ↑ CMAI, ABS, MMSE In afternoon, Teager score ↔ CMAI, ABS, MMSE In evening, Teager score ↑ CMAI, ABS, MMSE
	Time-stamped by observer	Friedman, *post-hoc* Wilcoxon signed-rank test	Teager score, in both morning, afternoon, and evening: ↑ during agitation, compared to pre-agitation and post-agitation
Goerss et al. (36)	CMAI	Spearman correlation	AMS showed: ↑ Total CMAI score ↑ CMAI physical sub-score ↑ CMAI physically nonaggressive sub-score ⊗ CMAI verbal sub-score ⊗ physically aggressive sub-score
	Real-time observation	Dirichlet regression	↓ observed apathy behaviour ↑ observed mannerism behaviour ↑ observed pacing behaviour
	Video observation	Dirichlet regression	→ observed apathy, mannerism, pacing behaviour
Ishimaru et al. (37)	CTSD, HADLS, MMSE, NPI-NH	Spearman correlation	Level of physical activity in daytime and full-day showed: ⊗ MMSE, CTSD, HADLS for moderate and severe DE ⊗ NPI-NH for moderate DE ↑ NPI-NH for severe DE
	NPI-NH Sub-score	Spearman correlation	Level of physical activity showed: ↑ Agitation/aggression sub-score in daytime and full-day ↑ night-time behaviour sub-score in full-day ⊗ other sub-scores
Knuff et al. (26)	High and low agitation groups classified by CMAI	Mann-Whitney U test	High agitation group had: ↑ Daytime and evening motor activity ⊗ Night-time motor activity
	CMAI, NPI, CSDD	Pearson correlation	Full-day, daytime, and evening motor activity was: ↑ CMAI total ↑ CMAI verbal agitation, non-aggressive physical agitation sub-score ↑ NPI total score ↑ NPI related items ⊗ CMAI aggressive physical agitation sub-score ⊗ CSDD total score Night-time motor activity was: ⊗ CMAI total, verbal agitation, non-aggressive and aggressive physical sub-score ⊗ NPI total score, related items, CSDD total score
Nagels et al. (38)	CMAI, MMSE	Spearman correlation	PIM, ZCM, TAT had: ↑ CMAI total score ↑ non-aggressive physical sub-score ⊗ aggressive physical behaviour, verbal agitation ZCM had: ↑ MMSE
Spasojevic et al. (39)	Two-step label by nurse charts and video recordings	SVM LR RF RF-C RF-RL	Performance of classifying agitation episodes (AUC): SVM: 0.595 LR: 0.590 RF: 0.624 RF-C: 0.632 RF-RL: 0.498
Valembois et al. (40)	NPI sub-scores	2-way ANOVA repeated measures	Motor activity level showed: ⊗ between agitation and no agitation in all timeslots ⊗ between anxiety and no anxiety in all timeslots ↑ with aberrant motor behaviour, compared to no aberrant motor behaviour from 9 am to 12 pm and 9 pm to 0 am ↓ with apathy, compared to no apathy from 9 am to 12 pm, 12 pm to 3 pm, 3 pm to 6 pm, and 6 pm to 9 pm.
Wijbenga et al. (41)	NPI-NH, CMAI, sleep time	Vector Autoregression	Motor activity (agitation) level showed: ↑ temporal relationship in sleep time in three out of five patients No association test with NPI-NH and CMAI

↑ Significant increase or higher or positively associated; ↓ Significant decrease or lower or negatively associated; → significant association/difference but direction (positive or negative, increase or decrease) not specified; ⊗, No significant difference or association.

ABS, Aggressive Behaviour Scale; AMS, Accelerometer Motion Score; ANOVA, Analysis of Variance; AUC, Area under Receiver Operating Characteristics Curve; CMAI, Cohen-Mansfield Agitation Inventory; CSDD, Cornell Scale for Depression in Dementia; CTSD, Cognitive Test for Severe Dementia; DE, dementia; HADLS, Hyogo Activities of Daily Living Scale; LR, Logistic Regression; MMSE, Mini Mental State Examination; NPI, Neuropsychiatric Inventory; NPI-NH, NPI – Nursing Home; PIM, Proportional Integrating Measure; RF, Random Forest; RF-C, RF with Cost; RF-RL, RF with Random Labels; SVM, Support Vector Machine; TAT, Time-Above-Threshold; ZCM, Zero Crossing Mode.

The Cohen-Mansfield Agitation Inventory (CMAI) and the Neuropsychiatric Inventory (NPI) were benchmarks for labelling the class or severity of the agitated behaviour completed by a proxy (51, 52). The CMAI is a 29-item questionnaire on agitated behaviour, on a seven-point scale of frequency. Its subgroup constructs or sub-scores are also commonly used, including the aggressive behaviour sub-score, the physical non-aggressive behaviour sub-score, and the verbally agitated behaviour sub-score (51). Both Knuff et al. (26) and Nagels et al. (38) divided the participants into those exhibiting high and low levels of agitation at a cut-off point of 50 for the CMAI total score. The NPI was developed to assess neuropsychiatric syndromes for patients with a score system calculated by multiplying the frequency (1 to 4 points) and severity (1 to 3 points) of each item on 10 domains. The instrument has been translated into different versions and languages (53), including a nursing home version (NPI–NH) used by Ishimaru et al. (37). The original version encompassed domains that could be broadly classified as depression-related or delusion-related items, while the sub-scores on anxiety, agitation, aberrant motor disturbance, apathy, and night-time disturbances appeared in our review. Other related instruments included the Aggressive Behaviour Scale (ABS) (54) and the Mini Mental State Examination (MMSE) (55).

In general, the application of wrist accelerometry (or movement measurements) in recognising potentially agitated individuals was supported by the significant association with the benchmark instrument CMAI (26, 35, 36, 38). In addition, during the occurrence of agitation episodes an increase in motor activity level was also reported by the wrist accelerometer (35). Interestingly, in a few studies a correlation with the non-aggressive agitation sub-score was demonstrated, but not with the aggressive agitation and verbal agitation sub-scores (26, 36, 38). In other words, high levels of motion were associated with non-aggressive manoeuvres, such as restlessness, mannerisms, pacing, and hiding, instead of with aggressive manoeuvres, such as hitting, grabbing, and kicking. The findings were also confirmed by real-time and video-recorded observations (36).

Six articles analysed the level of activity at different times. Some studies divided the day into daytime and night-time, while some split the day into morning, afternoon, evening, and night-time. During daytime, all studies reported significant associations between agitation and level of activity, while three out of four studies supported the association during evening. Mixed results were found in the afternoon and night-time. One reason for this could be the heterogeneity in time-splitting, measurement time, sampling, wearing time, and compliance, while confounding factors such as the environment and co-residence with other people could have influenced the level of activity during the night-time. In the afternoon, individuals in residential care homes may have some physical activities or entertainment arranged for them, while those in inpatient wards may have limited activities. These scheduled activities may have positive or negative impacts on people with dementia with agitation and other related behavioural constructs (56).

## Discussion and conclusion

Our review showed that the frequency or entropy (aggregated energy) of activity levels as measured by wrist accelerometers was associated with agitated behaviour in clinical settings despite differences in accelerometer devices and research protocols (26, 35, 36, 38). Nevertheless, one study reported poor discriminative performance from actigraphy in identifying agitation episodes (39). The objective, quantitative, real-time and prolonged nature of accelerometry could also complement the weaknesses of benchmark questionnaires or observations. However, improvement is required if accelerometers are to be used to identify or measure agitated behaviour episodes. Yet evaluations of the discriminating power of accelerometry on agitation or not are lacking, for example using sensitivity, specificity, or receiver-operating characteristic curves.

The time when behavioural disturbances were exhibited by agitated persons with dementia was commonly examined in clinical settings, with daytime and evening likely to be the most likely times. Au-Yeung et al. (34) also investigated the influence of environmental light, noise, temperature, humidity, and level of pollutants, in a study that employed wearable devices that monitored the skin temperature and heart rate of individuals (36). In one review, it was suggested that integrating accelerometers with other sensors may improve the accuracy with which agitation and aggressive behaviours are detected, and that more field validation is required (6). In short, multi-modal sensor platforms could provide clinical management with information that could be used to modify the environment and design entertainment programmes and activities, such as music interventions, to alleviate agitation and promote well-being among agitated individuals (57).

There are some drawbacks to wrist accelerometry. Accelerometry can only measure physical signals, and therefore cannot be used conduct an assessment of verbal agitation (58). Our review also found that the level of physical activity was not correlated with the verbal agitation construct in the questionnaire (26, 36, 38). The maintenance and power of the wearable devices could be another problem for 24-h surveillance. In fact, Bankole et al. (35) reported critical incidents that occurred in the study due to device break-down, albeit at a low rate. Discomfort in wearing the wrist accelerometers presented another notable problem, contributing to drop-outs, poor skin contact, and other compliance issues that were reported in the articles that we reviewed (37, 40). Some studies proposed using a depth-channel camera and machine learning model for behavioural surveillance as a compromise between privacy and the need to carry out monitoring (59, 60), and this has been successfully implemented in clinical settings (61). We also discovered some potential biases in the studies. For example, only records with high-quality data and sufficient time and days were analysed (39, 40). Agitated individuals might wear the devices less often and exhibit poorer compliance (26), while some agitated behaviour may result in poor contact with the wrist accelerometers and therefore lead to poor-quality data. Therefore, it is possible that only “obedient” agitated individuals or moments were included in the data.

There were some limitations to this review. First of all, selection bias could have resulted from the inclusion criteria, with only original articles and journal papers in English eligible for inclusion. Second, our review targeted clinical settings and discarded some studies with home-based or laboratory settings (62, 63). Those laboratory studies contributed more to the development of systems and classification models with more technical specifications than those studies that used secondary datasets or created data by instructing participants to perform agitated and non-agitated acts (64, 65). There were other data analysis methods, such as the inter-daily stability and intra-daily variability on actigraphic rest-and-activity rhythms (66). Some studies were also excluded because they were not focused on agitation, although they assessed the motor activity or rest patterns of the patients. In future studies, consideration may be given to investigating the application of accelerometry to evaluate interventions such as those involving medications (67, 68), electrical stimulation (69), and light therapy (70).

Given that agitation is common among people with dementia, employing technology for assessing the agitated behaviour may be beneficial for both patients and caregivers. In the future, we recommended standardising the protocol for assessing agitated behaviour by unifying the parameter-of-interest, cut-offs, measurement period, and sampling window for wrist accelerometry/actigraphy. Validating multi-modal sensors, which measure different parameters may be the next step to increase the accuracy of agitated behaviours assessment.

## Author contributions

DC and KH designed the study. JC, BS, and DW searched for and screened studies. JC and BS extracted data from the selected studies. JC, DW, DC, and AL wrote the manuscript. KH critically reviewed it. All authors reviewed and edited the manuscript.

## Conflict of interest

The authors declare that the research was conducted in the absence of any commercial or financial relationships that could be construed as a potential conflict of interest.

## Publisher's note

All claims expressed in this article are solely those of the authors and do not necessarily represent those of their affiliated organizations, or those of the publisher, the editors and the reviewers. Any product that may be evaluated in this article, or claim that may be made by its manufacturer, is not guaranteed or endorsed by the publisher.
