# Supplementary material for: Wrist accelerometry for monitoring dementia agitation behaviour in clinical settings: A scoping review
Source: Front Psychiatry. 2022 Sep 16;13:913213. doi: 10.3389/fpsyt.2022.913213 (PMC9523077; doi:10.3389/fpsyt.2022.913213)
Supplement: Supplementary file 1 [file Table_1.DOCX]

# Supplementary Table 1. Full systematic search strategy for the literature review.

| Database | Dementia-related keywords | Accelerometry-related keywords | Agitation-related keywords |
| --- | --- | --- | --- |
| PubMed | Dementia OR Alzheimer Disease OR Cognitive Dysfunction OR Lewy Body Disease OR Memory Disorder [MH] | (Acelerometry OR Wearable Electronic Devices OR Machine Learning OR Ambulatory Monitoring [MH]) OR (“motion sens*”[Title/Abstract] | (Psychomotor Agitation OR Wandering Behavior [MH]) OR (Aggression OR Agitation OR Irritability OR Sundowning OR Challenging Behavior OR Challenging Behaviour [All]) |
| CINAHL | (Dementia+ OR Dementia, Vascular+ OR Delirium, Dementia, Amnestic, Cognitive Disorders+ OR Lewy Body Disease OR Amnesia+ OR Mild Cognitive Impairment [MH]) OR Alzhemier’s Disease [MM] OR “Dementia” | Accelerometry+ OR Wearable Sensors+ OR Machine Learning+ [MH] | (Agitation OR Wandering Behavior [MM]) OR (Psycholomtor Agitation+ OR Aggression+ OR Violence+ [MH]) OR (sundowning OR challenging behaviour OR challenging behavior) |
| PsycInfo | Dementia OR Cognitive Impairment OR Amnesia OR Mild Cognitive Impairment OR Dementia with Lewy Bodies OR Alzheimer’s Disease [MH] | (Machine Learning OR Wearable Device [MH]) OR (Accelerometry OR accelerometry [NOFT]) | (Agitation OR Wandering Behavior [MH]) OR (aggression OR violence OR sundowning OR challenging behaviour OR challenging behavior [NOFT]) |
| Web of Science | (Dementia* OR alzheimer* OR "cognitive dysfunction" OR "cognitive decline" OR "cognitive impairment" OR lewy OR amnesi* OR "memory disorder*" [TS]) | (acceleromet* OR "wearable sens*" OR "wearable electronic" OR "machine learning" OR "wrist band" OR actigraph* OR "remote monitoring" OR gyroscope* OR goniomet* OR "step count" OR "fitness tracker*" OR "behavio*r monitor*" [TS]) | (agitat* OR aggressi* OR sundowning OR wandering OR restless* OR "challenging behavio*r" OR irritability OR violence OR "problem* behavio*r" [TS]) |
| EMBASE | (Dementia* OR alzheimer* OR "cognitive dysfunction" OR "cognitive decline" OR "cognitive impairment" OR lewy OR amnesi* OR "memory disorder*" [ti, ab, kw]) | (acceleromet* OR "wearable sens*" OR "wearable electronic" OR "machine learning" OR "wrist band" OR actigraph* OR "remote monitoring" OR gyroscope* OR goniomet* OR "step count" OR "fitness tracker*" OR "behavio*r monitor*" [ti, ab, kw]) | (agitat* OR aggressi* OR sundowning OR wandering OR restless* OR "challenging behavio*r" OR irritability OR violence OR "problem* behavio*r" [ti, ab, kw]) |

Remark: the topic “actigraphy” is under the Mesh Term of accelerometry
